# Comparison of the Radiographic and Clinical Outcomes between the Sinus Tarsi and Extended Lateral Approaches for Intra-Articular Calcaneal Fractures: A Retrospective Study

**DOI:** 10.3390/jpm14030259

**Published:** 2024-02-28

**Authors:** Jui-Ting Mao, Chien-Ming Chen, Chung-Wei Lin, Hsuan-Lun Lu, Chien-Chung Kuo

**Affiliations:** 1 Department of Orthopedics, China Medical University Hospital, Taichung 40447, Taiwan; jtmao0911@gmail.com (J.-T.M.); 023325@tool.caaumed.org.tw (C.-M.C.); visionaryshade@gmail.com (C.-W.L.); 2 Department of Orthopedics, School of Medicine, China Medical University, Taichung 40447, Taiwan; 3 Department of Biomedical Engineering, Da-Yeh University, Changhua 51591, Taiwan; hllu@mail.dyu.edu.tw

**Keywords:** extended lateral approach, sinus tarsi approach, intra-articular calcaneal fractures

## Abstract

The aim of this study was to compare the radiological and functional outcomes of the extended lateral and sinus tarsi approaches for managing displaced intraarticular calcaneal fractures. This retrospective study involved 44 patients with displaced intra-articular calcaneal fractures. The patients were treated with either the extended lateral or sinus tarsi approach and followed up for at least a year. The radiological and clinical outcomes were compared between the approaches. The waiting time for surgery was shorter and the complication rate was lower in the sinus tarsi approach group than in the other group. There were no significant differences in the American Orthopedic Foot and Ankle Society ankle–hindfoot score, Foot Function Index, or visual analog scale score between the groups. In both groups, the radiological outcomes (Böhler angle, calcaneal width, and calcaneal height) were better postoperatively than preoperatively. The sinus tarsi approach is a safe and effective alternative to the extended lateral approach for managing displaced intraarticular calcaneal fractures. It is associated with a lower complication rate and a shorter waiting time for surgery than the extended lateral approach, with similar functional and radiological outcomes.

## 1. Introduction

Calcaneal fractures constitute approximately 60% of all tarsal fractures and are typically caused by falls from heights or motor-vehicle accidents [1,2,3]. Displaced intra-articular fractures account for 60–75% of all calcaneal fractures [4]. These fractures often lead to soft tissue injuries and a loss of function in the subtalar joint area [3]. However, the treatment of calcaneal fractures can be challenging for surgeons. The conservative management of these fractures is associated with a poor prognosis, with patients progressing to subtalar joint arthritis, malunion, and poor functional outcomes [5]. Therefore, open reduction and internal fixation are recommended for managing displaced intra-articular calcaneal fractures [6,7].

The extensile lateral approach (ELA) is considered the gold standard for calcaneal fractures [8,9,10]. However, despite adequate exposure, the ELA is associated with a high incidence of postoperative complications, such as skin edge necrosis, wound infection, and nonunion [11,12]. The sinus tarsi approach (STA), a minimally invasive technique, has recently gained popularity for reducing the complications associated with calcaneal fracture treatment. The purpose of this study was to compare the radiological and clinical functional outcomes of the ELA and STA to add to the existing literature on calcaneal fracture management.

## 2. Materials and Methods

### 2.1. Patient Population

**Figure 1:**
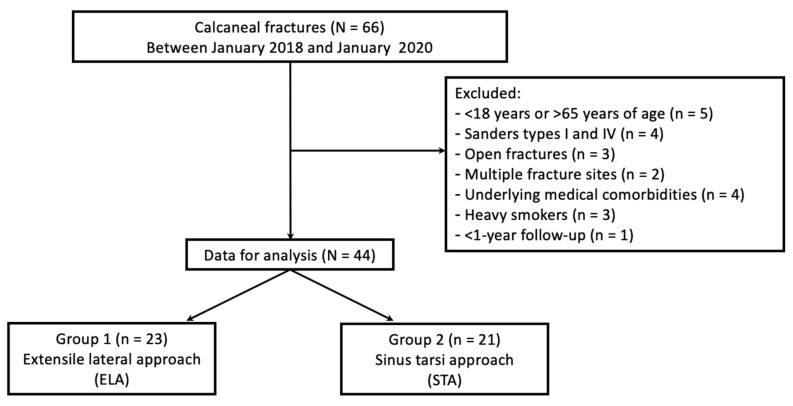
Participant enrollment flow chart. ELA: extensile lateral approach; STA: sinus tarsi approach.

Between January 2018 and January 2020, 44 patients with displaced intra-articular calcaneal fractures (Sanders types II and III) were treated by two experienced surgeons at a level-1 trauma center. Each of the two surgeons performed the ELA and STA separately. All patients were 18–65 years old; they had sustained injuries from falls or motor-vehicle accidents and had undergone open reduction and internal fixation with either the ELA or STA. Patients were excluded if they were heavy smokers (>20 cigarettes per day); had an open fracture, multiple fracture sites, or underlying medical comorbidities (all with Type 2 diabetes); and had been followed up for less than 1 year (Figure 1). The sex distribution did not differ significantly between the groups (Group 1: 78.3% men and 21.7% women; Group 2: 90.5% men and 9.5% women; *p* = 0.416). Furthermore, the two groups did not differ significantly in terms of the mean patient age (Group 1: 45.30 ± 10.00 years; Group 2: 42.86 ± 12.95 years; *p* = 0.581) and mean BMI (Group 1: 24.72 ± 4.20 kg/m^2^; Group 2: 24.93 ± 3.84 kg/m^2^; *p* = 0.7). The fracture types included Sanders types II and III, and their proportions did not differ significantly between the groups (Table 1).

### 2.2. Surgical Technique

The patients were placed in the lateral decubitus position under spinal anesthesia using a tourniquet. In both groups, all patients were administered prophylactic antibiotic therapy with first-generation cephalosporin, both preoperatively and postoperatively; the postoperative course comprised three doses spaced 8 h apart.

#### 2.2.1. Group 1 (ELA)

The standard ELA described by Benirschke and Sangeorzan in 1993 was used to expose the subtalar joint and lateral wall [13]. After making an incision directly into the bone, a subperiosteal flap was raised and held using 1.6 mm Kirschner wires. Following the direct reduction of the fracture site, locking calcaneal plates (Acumed Combo Calcaneal plate) were used for fixation.

#### 2.2.2. Group 2 (STA)

**Figure 2:**
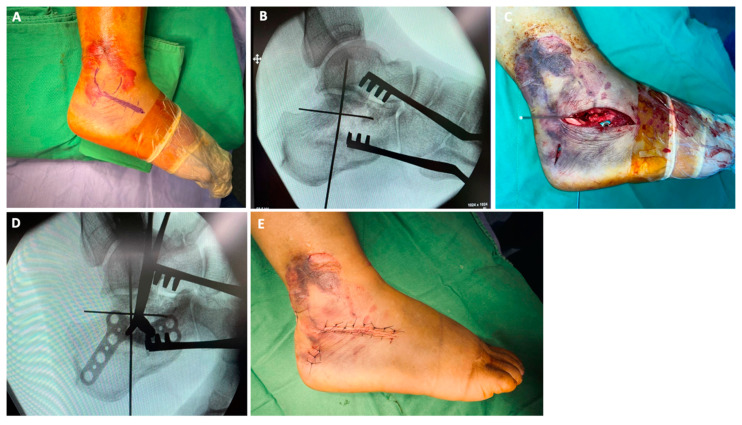
Two-incision STA for a right DIACF in a 37-year-old woman. (**A**) Skin incision in the STA. (**B**) Reduction and fixation with Kirschner wires. (**C**) Application of a locking plate to fix the calcaneus through two incisions. (**D**) Confirmation of adequate plate positioning under fluoroscopy. (**E**) Removal of stitches 2 weeks after operation. DIACF: displaced intra-articular calcaneal fracture; STA: sinus tarsi approach.

The STA used in this study was based on the technique described by Chiang et al. in 2021 [14]. The first incision, approximately 3–4 cm in length, was made horizontally along the tip of the lateral malleolus to a level distal to the calcaneocuboid joint. The incision allowed for adequate exposure of the subtalar joint from the calcaneocuboid joint to the posterior facet of the calcaneus. The peroneal tendons were identified and pulled inferiorly. A reduction was achieved and temporarily fixed using Kirschner wires. Another incision was made in the posterior calcaneal tuberosity. A locking plate (Acumed Combo Calcaneal plate) was used to fix the calcaneus through these two incisions (Figure 2).

### 2.3. Postoperative Protocol

Dressings were changed regularly to monitor the wound condition, and stitches were removed 2 weeks postoperatively if there was no surgical site infection. Patients in both groups started active and passive range motion exercise regimes for the foot and ankle joints immediately after the surgery, based on their tolerance. Partial weight bearing was initiated at 8 weeks postoperatively, and full weight bearing was allowed at 12 weeks postoperatively once radiographic union was achieved. We determined radiographic union by regularly following up with X-rays to monitor fracture healing. Radiographic union was defined based on radiological findings such as callus formation and cortical bridging.

### 2.4. Variables, Data Sources, and Outcome Assessment

All patients were followed up for at least 1 year. Postoperatively, radiographs were obtained at the 1-month, 3-month, 6-month, and 1-year follow-ups. The data obtained from the medical records included basic demographics (age, sex, body mass index [BMI], and etiology), Sanders type, waiting time for surgery, blood loss, operation time, and complication rates. The clinical outcomes included wound complications, the American Orthopedic Foot and Ankle Society (AOFAS) ankle–hindfoot score, the Foot Function Index (FFI), and the visual analog scale (VAS) score. Radiological assessments comprised an evaluation of the reduction quality of the articular surface and measurements of the Böhler angle (measured between a line drawn from the highest point of the anterior process and posterior articular facet and another line joining the highest point of the posterior articular facet with the highest point of the calcaneal tuberosity), calcaneal width, and calcaneal height.

Complications were categorized as minor or major. Minor complications, including superficial infections, were defined as those that were treated without reoperation. Major complications were defined as deep infections that required surgical intervention. The AOFAS ankle–hindfoot score, FFI, and VAS score were self-assessed by a single resident doctor for each patient 1 year after surgery to determine their clinical outcomes. Articular surface anatomic reduction was defined as no obvious displacement in an X-ray. Nearly anatomic reduction was defined as a displacement between 0 mm and 2 mm, and poor reduction was defined as a displacement of >2 mm among the reduction qualities.

### 2.5. Statistical Analysis

This study was approved by the institutional ethical review board (CMUH111-REC3-132). We conducted a power analysis using G*Power to ensure that the sample size was adequate for comparing the ELA and STA. According to the G*Power analysis, when the power was >0.8, the number of cases required was 18 for each group. Therefore, our case number is sufficient for the analysis. Dichotomous data were compared between the groups using Fisher’s exact test. Differences in the measured variables were determined using Mann–Whitney U tests. The treatment effects were analyzed using Wilcoxon signed-rank tests. The statistical significance was set at *p* < 0.05. Data analyses were performed using SPSS ver. 19.0 (SPSS Inc., Chicago, IL, USA).

## 3. Results

The waiting time for surgery was significantly shorter in Group 2 than in Group 1 (3.76 ± 1.81 days vs. 10.78 ± 2.76 days, *p* < 0.05). However, Groups 1 and 2 did not differ significantly in terms of the operation time (131.00 ± 49.34 min vs. 129.38 ± 37.49 min, *p* = 0.689), blood loss volume (43.70 ± 42.91 mL vs. 24.05 ± 16.25 mL, *p* = 0.091), and hospital stay duration (6.22 ± 4.17 days vs. 5.19 ± 1.94 days, *p* = 0.867) (Table 2).

The postoperative radiographic parameters (Böhler angle, calcaneal width, and calcaneal height) significantly improved compared to the corresponding preoperative values (Table 3).

Both groups achieved a 100% union rate, with no significant differences in the union time between them (Group 1: 86.96 ± 20.32 days; Group 2: 87.14 ± 21.48 days; *p* = 0.806; Table 4). No significant differences were detected in the Böhler angle or the reduction quality of the articular surface between the groups. Furthermore, the functional scores (comprising the AOFAS ankle–hindfoot score, FFI, and VAS score) did not differ significantly between the groups at the 1-year follow-up (Table 4).

Patients with minor complications required antibiotic treatment and wound care. For major complications, patients underwent debridement for infection control, fortunately without the need for implant removal. The overall complication rate was significantly higher in Group 1 than in Group 2 (66.67% vs. 33.52%, *p* < 0.05). Six of the eight patients with infections were from Group 1; the remaining two were from Group 2. Among the patients with infections, three in Group 1 had major complications (Table 5).

## 4. Discussion

In this study, we aimed to retrospectively compare the short-term outcomes of displaced intraarticular calcaneal fractures treated with the ELA or STA. Our results suggested that patients who underwent the STA experienced shorter waiting times for surgery and lower complication rates. There were no significant differences in the functional and radiological outcomes between the approaches.

In 2008, Weber et al. conducted the first comparative study on the ELA and STA in 50 patients; they compared the outcomes of displaced intraarticular calcaneal fractures treated using the ELA with lateral plate fixation and the STA with cannulated screw fixation. Their study revealed a shorter operative time with the STA; however, no significant differences in the complication rate, clinical outcomes, and radiographic results were noted between the approaches [15]. In 2013, Kline et al. performed a study on 112 patients; the overall complication rates differed significantly between the ELA and STA groups (29% vs. 6%). However, the functional and radiographic outcomes were similar between the groups [16]. In both studies, screw fixation was performed in the STA group.

Kir et al. compared cannulated screw and miniplate fixation treatments using the STA in 60 patients with Sanders type II and III calcaneal fractures and reported better functional outcomes and a lower reoperation rate with miniplate fixation [17]. As plate fixation has better outcomes than other methods, several studies have used locking plate fixation via the STA approach. Xia et al. compared the outcomes of 49 and 59 patients treated with the ELA and STA, respectively; the fractures in both groups were fixed with plates. There were no significant differences in the radiographic outcomes between the groups; however, the STA group had a shorter operative time and fewer postoperative wound complications [18]. Basile et al. conducted another comparative study on 38 patients and reported similar clinical and radiographic outcomes between the STA and ELA groups; however, the STA group experienced a significantly faster surgical procedure and shorter waiting time for surgery. Although a lower wound complication rate was observed in the STA group, the difference was not significant [19]. Chiang et al. reported a modified two-incision STA with plate fixation and demonstrated its safety and effectiveness [14]. Our study is the first to compare the two-incision STA with the ELA.

Despite surgical intervention for intraarticular calcaneal fractures, subtalar arthritis may develop. Herscovici et al. reported on thirty-five patients who underwent surgery for a calcaneal fracture, after which posttraumatic subtalar arthritis developed in twelve patients [20]. The higher incidence of wound complications after calcaneal fracture treatment with the ELA is because wound healing depends on the subperiosteal flap covering the lateral wall, which is thin and vulnerable [21,22]. Surgery via the ELA is safe when the skin shows a positive wrinkle sign without pitting edema [23]. In our study, the mean waiting time for the ELA was 10.78 days. The STA causes less damage to the subperiosteal flap and requires less demanding skin conditions. The mean waiting time for surgery with the STA was 3.76 days. However, the STA has some limitations, including poor exposure of the fracture site and difficulties in accessing the fracture for reduction [16,24]. In our opinion, early intervention, standard calcaneal plate insertion via a two-incision wound, and arthroscopic-assisted reduction could overcome these limitations. Our study also had some limitations, including its retrospective design and small sample size. In addition, the follow-up period was limited to 12 months, which may not fully capture the long-term outcomes of the intervention under investigation. Future studies should address these limitations with a larger sample and longer follow-up.

## 5. Conclusions

In conclusion, the STA demonstrated lower rates of wound complications and shorter waiting times for surgery than the ELA while achieving similar functional and radiological outcomes in the treatment of displaced intra-articular calcaneal fractures. Therefore, we recommend using the STA to treat these fractures.

## Figures and Tables

**Table 1:** Patient characteristics.

	ELA	STA	*p*
Number of patients	23	21	
Mean age (years)	45.30 ± 10.00	42.86 ± 12.95	0.581
Sex			0.416
Male	18 (78.3%)	19 (90.5%)	
Female	5 (21.7%)	2 (9.5%)	
BMI (kg/m^2^)	24.72 ± 4.20	24.93 ± 3.84	0.7
Etiology			0.489
Falls	21	21	
Traffic accidents	2	0	
Sanders type			0.880
Type IIA	3	4	
Type IIB	6	5	
Type IIC	4	3	
Type IIIAB	2	1	
Type IIIAC	1	2	
Type IIIBC	0	0	

BMI: body mass index; ELA: extensile lateral approach; STA: sinus tarsi approach.

**Table 2:** Perioperative period.

	ELA	STA	*p*
Waiting time for surgery (days)	10.78 ± 2.76	3.76 ± 1.81	<0.05
Operation time (min)	131.00 ± 49.34	129.38 ± 37.49	0.689
Blood loss (mL)	43.70 ± 42.91	24.05 ± 16.25	0.091
Hospital stay (days)	6.22 ± 4.17	5.19 ± 1.94	0.867

ELA: extensile lateral approach; STA: sinus tarsi approach.

**Table 3:** Radiographic parameters.

	Preoperative	Postoperative	*p*
**ELA**			
Böhler angle (°)	11.27 ± 9.01	28.10 ± 5.10	<0.001
Calcaneal width (cm)	5.14 ± 0.48	4.02 ± 0.45	<0.001
Calcaneal height (cm)	5.16 ± 0.51	5.90 ± 0.39	<0.001
**STA**			
Böhler angle (°)	10.75 ± 6.88	29.89 ± 5.44	<0.001
Calcaneal width (cm)	5.10 ± 0.57	4.09 ± 0.42	<0.001
Calcaneal height (cm)	5.16 ± 0.47	5.78 ± 0.42	<0.001

ELA: extensile lateral approach; STA: sinus tarsi approach.

**Table 4:** Radiographic and clinical outcomes.

	ELA	STA	*p*
Union rate	100%	100%	
Union time (days)	86.96 ± 20.32	87.14 ± 21.48	0.806
Bohler angle (Pre) (°)	11.27 ± 9.01	10.75 ± 6.88	0.953
Bohler angle (Post) (°)	28.10 ± 5.10	29.89 ± 5.44	0.329
Reduction quality			0.228
Anatomic	12 (52.17%)	15 (71.43%)	
Nearly anatomic	11 (47.83%)	6 (28.57%)	
VAS pain score	0.74 ± 0.69	0.81 ± 0.75	0.779
FFI	18.78 ± 9.93	17.67 ± 8.14	0.906
AOFAS score	81.26 ± 6.02	81.10 ± 5.80	0.887

AOFAS: American Orthopedic Foot and Ankle Society; ELA: extensile lateral approach; FFI: Foot Function Index; Pre: preoperative; Post: postoperative; STA: sinus tarsi approach; VAS: visual analog scale.

**Table 5:** Complications.

	ELA 23	STA 21	*p*
Overall complications	14 (60.87%)	5 (23.81%)	<0.05
Subtalar arthrosis	5 (21.74%)	3 (14.29)	
Superficial infection	6 (26.09%)	2 (9.52%)	
Deep infection	3 (13.04%)	0	

ELA: extensile lateral approach; STA: sinus tarsi approach.

## Data Availability

Further data that support the reported results are available from the corresponding author upon reasonable request.
